# Detection and characterization of genome-wide mutations in M1 vegetative cells of gamma-irradiated Arabidopsis

**DOI:** 10.1371/journal.pgen.1009979

**Published:** 2022-01-20

**Authors:** Satoshi Kitamura, Katsuya Satoh, Yutaka Oono

**Affiliations:** Project “Ion Beam Mutagenesis”, Department of Radiation-Applied Biology Research, Takasaki Advanced Radiation Research Institute, National Institutes for Quantum Science and Technology, Takasaki, Japan; Tsinghua University, CHINA

## Abstract

Radiation-induced mutations have been detected by whole-genome sequencing analyses of self-pollinated generations of mutagenized plants. However, large DNA alterations and mutations in non-germline cells were likely missed. In this study, in order to detect various types of mutations in mutagenized M1 plants, anthocyanin pigmentation was used as a visible marker of mutations. Arabidopsis seeds heterozygous for the anthocyanin biosynthetic genes were irradiated with gamma-rays. Anthocyanin-less vegetative sectors resulting from a loss of heterozygosity were isolated from the gamma-irradiated M1 plants. The whole-genome sequencing analysis of the sectors detected various mutations, including structural variations (SVs) and large deletions (≥100 bp), both of which have been less characterized in the previous researches using gamma-irradiated plant genomes of M2 or later generations. Various types of rejoined sites were found in SVs, including no-insertion/deletion (indel) sites, only-deletion sites, only-insertion sites, and indel sites, but the rejoined sites with 0–5 bp indels represented most of the SVs. Examinations of the junctions of rearrangements (SVs and large deletions), medium deletions (10–99 bp), and small deletions (2–9 bp) revealed unique features (i.e., frequency of insertions and microhomology) at the rejoined sites. These results suggest that they were formed preferentially via different processes. Additionally, mutations that occurred in putative single M1 cells were identified according to the distribution of their allele frequency. The estimated mutation frequencies and spectra of the M1 cells were similar to those of previously analyzed M2 cells, with the exception of the greater proportion of rearrangements in the M1 cells. These findings suggest there are no major differences in the small mutations (<100 bp) between vegetative and germline cells. Thus, this study generated valuable information that may help clarify the nature of gamma-irradiation-induced mutations and their occurrence in cells that develop into vegetative or reproductive tissues.

## Author summary

Mutations that occur in plant genome are not only related to plant evolution and speciation in nature, and also useful to identify novel gene functions and to develop new cultivars. Ionizing radiations induce various types of mutations throughout genomes in individual cells of an irradiated/mutagenized plant. However, current knowledge on radiation-induced genome-wide mutations in plants relied on the analyses of self-pollinated generations (M2 or later generations) of the mutagenized plants (M1 generation). Thus, mutations that are hardly transmitted to the next generation and those occurred in non-germline cells could not be investigated. Here, using anthocyanin pigmentation as a visible marker to reduce the genomic complexity in M1 plants, we achieved reliable detection of radiation-induced genome-wide mutations. We demonstrated that rearrangements, which were hardly detected in previous analyses using M2 genomes, occurred substantially often in gamma-irradiated M1 cells. We also revealed that mutation profile of the M1 cells was comparable with that of M2 genomes reported in previous analyses, except for the higher proportion of rearrangements in the M1 genome. Together with unique features at rejoined sites of rearrangements, medium deletions, and small deletions in the M1 genome, our findings are helpful to know the nature of genome-wide mutations induced by gamma-irradiation.

## Introduction

Because plants are sessile organisms, they are constantly exposed to harmful factors, including pathogens, toxic chemicals, ultraviolet light, and ionizing radiation. These factors can damage genomic DNA in various ways, leading to oxidized bases, abasic sites, single-strand breaks, and double-strand breaks (DSBs). To cope with various DNA lesions, plants, like all living organisms, have evolved several DNA repair systems. For example, DSBs, which are the most serious type of DNA damage, are repaired by two main pathways, namely homologous recombination, which is possible if there are homologous sequences around the DSBs, and non-homologous end-joining (NHEJ), which may occur at any point in the cell cycle [1]. Despite these intrinsic repair systems, DNA lesions are often incompletely repaired, and those that remain may result in permanent genomic mutations. Additionally, some DNA lesions are bypassed by error-prone polymerases during DNA replication, leading to mutations [2]. Genetic mutations may alter gene functions, which can influence various biological processes, including speciation, evolution, and breeding. Therefore, identifying and characterizing mutations is important for clarifying fundamental mechanisms broadly related to plant biology.

Chemical and physical mutagens have been used for mutagenesis experiments. Because ionizing radiation, such as X-rays and gamma-rays, can induce various types of mutations throughout genomes without targeting specific sequences, they are considered to be good mutagens for research and breeding purposes. Recent technological progress in plant science has resulted in the use of next-generation sequencing (NGS) instead of classical mutation analyses [3,4]. This has enabled investigations of the mutation profiles of whole genomes, especially for species with relatively small genomes, including Arabidopsis and rice. In Arabidopsis, spontaneous mutations [5,6] as well as artificial mutations induced by fast neutrons [7], accelerated ions [8–10], and gamma-rays [11] have been revealed by whole-genome sequencing (WGS). These earlier studies determined the mutation frequencies and spectra induced by those mutagens. Although WGS is a powerful tool for analyzing genome-wide mutations qualitatively and quantitatively, mutation analyses by WGS have been performed exclusively with the M2 generation (the second generation of mutagenized plants produced via self-pollination) or a later generation in which induced mutations are either homozygous or heterozygous for all loci. However, detecting genomic mutations in the M1 generation is warranted because the genome of this generation reflects the direct effects of mutagens and it harbors all types of mutations, including large deletions and chromosomal rearrangements, which may not be transmitted to the next generation. Additionally, there is no information regarding the genome-wide mutation profiles in M1 except for the clues provided by the results of analyses of M2 or later generations. Accordingly, it is difficult to discuss the similarities/differences in the occurrence of mutations in different cell types (e.g., germline or somatic cells) [12]. The most prominent hindrance to genomic analyses of M1 is the extensive chimerism in this generation. When exposed to some mutagenic conditions, individual cells of an organism are independently affected and they divide to form cell populations with a unique genomic composition. If methods are developed to resolve this chimerism, genomic analyses of M1 might be possible.

Some recombinant marker genes have been exploited for detecting certain types of mutations in M1 plant genomes [13,14]. For example, the GUS (β-glucuronidase) gene can be used to visualize mutated cells as blue spots *in planta* following the restoration of a nonfunctional GUS gene [15]. Markers containing an artificially inducible DSB site (i.e., I-*Sce*I and Cas9 nucleases) have also been developed [16,17]. Such a system facilitates thorough examinations of the process involved in repairing a DSB in a transgene. Thus, it has clarified the DSB repair mechanisms in plants. For example, in plant somatic cells, DSBs are mainly repaired via classical NHEJ (c-NHEJ), which is initiated by the attachment of Ku70/Ku80 heterodimers to DSB ends. In Ku-depleted conditions, DSBs are repaired via alternative end-joining (alt-EJ) in which DSBs are rejoined by annealing the short stretches of complementary homologous sequences (microhomology; MH) at both ends of the DSB [18–20]. However, there are limitations to the utility of these systems for detecting mutations throughout a genome, making it difficult to comprehensively analyze genomic mutations.

Plant pigments are often used as visual markers for detecting mutations [21,22]. Anthocyanins, which are a subclass of flavonoid pigments, are responsible for the red, purple, or blue coloration of aerial plant parts, such as the leaves, stems, flowers, and fruits. The genes associated with anthocyanin biosynthesis are conserved in the plant kingdom [23]. Moreover, unlike chlorophylls, a lack of anthocyanins does not result in non-viable cells. In Arabidopsis, the anthocyanin biosynthetic pathway has been fully elucidated at the molecular level [24]. The red coloration of Arabidopsis vegetative tissues is due to anthocyanins, and such pigmentation is mediated by a single enzymatic pathway (S1 Fig). Because each enzyme catalyzing a step of the pathway is encoded by a single gene, loss-of-function mutations to even one of these genes result in a lack of red pigmentation. Therefore, we intended to use this simple and robust intrinsic pathway to directly detect the decreased chimerism in mutagenized M1 plants. In this study, we exploited the loss of heterozygosity (LOH) of three anthocyanin biosynthetic genes in Arabidopsis. The triple heterozygous seeds were irradiated with gamma-rays. The resulting M1 seedlings were examined regarding the accumulation of anthocyanins in their aerial tissues. Genomic DNA was extracted from the anthocyanin-less sectors, which contained cells with genomes derived from only a few ancestral cells in the shoot apical meristem (SAM) of the irradiated embryo. This genomic DNA was used for an NGS analysis, with a coverage depth of up to approximately 100×, making it possible to reliably characterize the mutations induced by gamma-rays in the M1 genome.

## Results

### Isolation of anthocyanin-less sectors from irradiated triple heterozygous Arabidopsis seedlings

**Fig 1:**
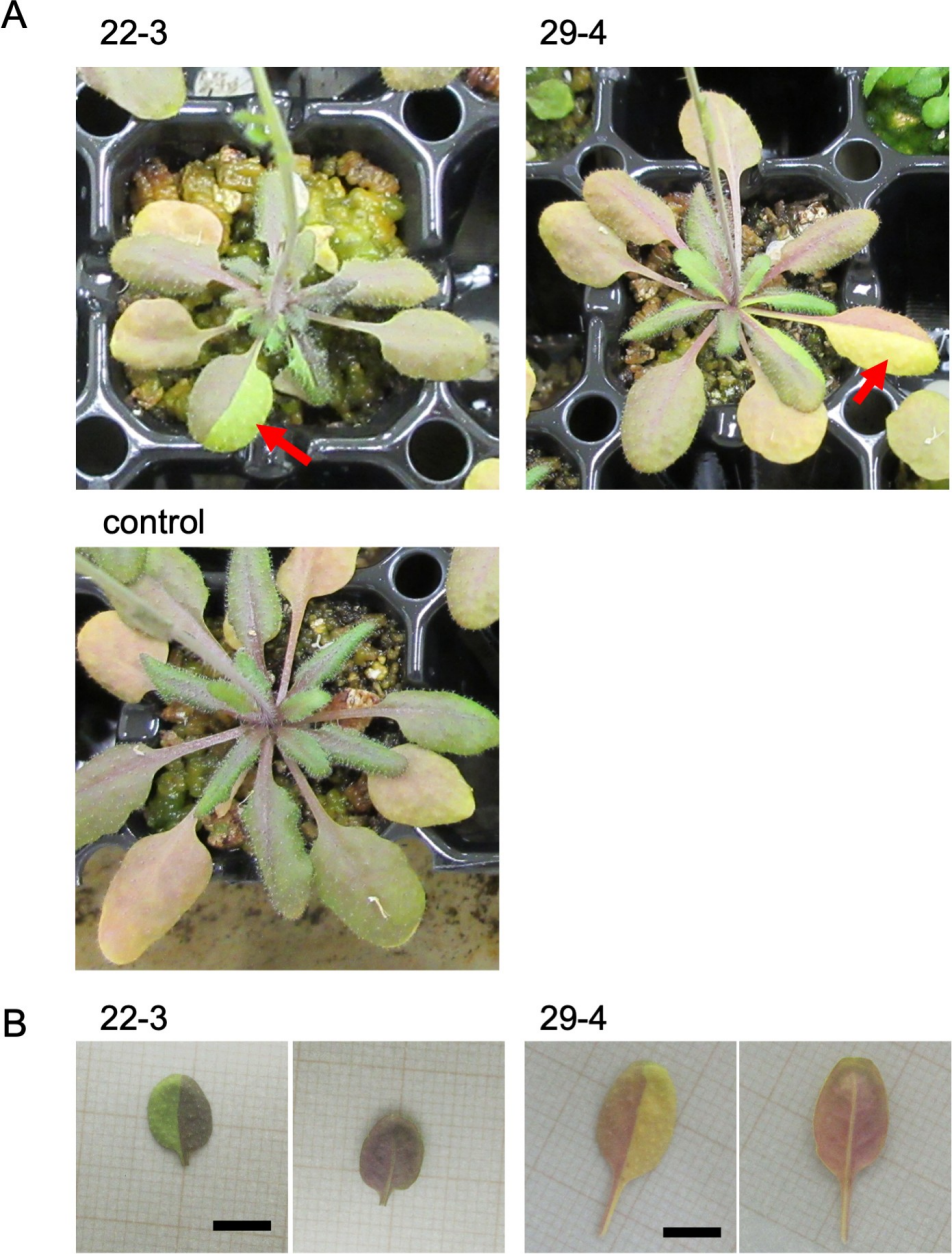
Anthocyanin-less sectors in triple heterozygous plants irradiated with gamma-rays. (A) Vegetative tissues of 22–3 (top left) and 29–4 (top right) plants. Red arrows indicate anthocyanin-less sectors analyzed by NGS. A non-irradiated control plant (bottom left) is also shown. These plants were grown in 100 mm^2^ pots. (B) Adaxial (left) and abaxial (right) surfaces of leaves indicated by an arrow in (A). Bars indicate 5 mm.

To accurately detect whole genome mutations in M1 plants, cell populations or tissues with minimal chimerism are required. Thus, the LOH associated with the anthocyanin accumulation phenotype in aerial tissues was used to obtain such low chimeric tissues from M1 plants. Triple mutants, in which three anthocyanin biosynthetic genes (*TT4*, *TT3*, and *TT18*) were mutated (S1 Fig), were crossed with wild-type Columbia. The resulting F1 seeds that were heterozygous for the three anthocyanin genes (genotype: *TT4tt4 TT3tt3 TT18tt18*) were irradiated with gamma-rays (1,000 Gy). The dose corresponded to half of the shoulder dose for a survival curve of the irradiated seeds [11]. Seedlings that germinated from the irradiated and non-irradiated seeds were grown under normal conditions and analyzed regarding the anthocyanin accumulation in aerial tissues. In a few irradiated plants, sectors lacking anthocyanin pigmentation were detected in vegetative tissues (Fig 1A). Of the 163 analyzed M1 plants, anthocyanin-less sectors were detected in seven plants (approximately 4% of the irradiated plants) (S2 Fig). The anthocyanin-less sectors were in the eighth or subsequent leaves. Of these seven plants, five had anthocyanin-less sectors on two or more leaves. Anthocyanin-less reproductive tissues, such as flower sepals, siliques, and seed coat, were not detected in these seven plants or in any of the other M1 plants. Interestingly, in all seven plants, although the adaxial surface was indeed anthocyanin-less, red pigmentation was observed on the abaxial surface, suggestive of the presence of anthocyanin (Fig 1B). Sectors lacking anthocyanin pigmentation were not detected in 168 non-irradiated plants, implying these sectors were the result of gamma irradiation. The anthocyanin-less sectors, including the anthocyanin-positive abaxial side, were excised for a DNA extraction. The purified DNA was used for WGS, which was conducted to clarify the mutational characteristics of the genomes in the cells of the sectors.

### Whole-genome sequencing of the anthocyanin-less sectors

If the DNA materials are from a single cell population with the same mutations, every mutated locus should include a wild-type allele and a mutant allele (50% of each) because each radiation-induced mutation occurs in either one of two sets of chromosomes. The M1 tissues, however, likely contain multiple cell populations and the wild-type:mutant allele ratio at every locus is expected to theoretically be less than 0.5; the exact ratio will depend on the extent of the chimerism. For example, if cells with certain mutations represent about a third of the cells in the sector, then allele frequencies (AFs) for these mutations will theoretically be 0.17 (i.e., 0.5 × 0.33). Although the sectors used in this study mainly consisted of single-cell-derived populations exhibiting an anthocyanin-less phenotype, the abundance of these cells could not be determined based solely on the phenotype. Therefore, to correctly detect mutations with a relatively low AF, we obtained more NGS reads than is required for standard mutation analyses of M2 or later generations, in which the AF is theoretically 1.0 (homozygous) or 0.5 (heterozygous) for every locus. In this study, using DNA independently extracted from seven anthocyanin-less sectors, approximately 620 million reads were obtained following 150-bp paired-end NGS sequencing. After removing low-quality, unpaired, and duplicated reads, the remaining clean reads were mapped to the Arabidopsis reference genome. The coverage depth per sector was 72× to 97× (average of 85×; S1 Table), which is about 2 or more times greater than the coverage depth of recent analyses of the Arabidopsis M2 genome [9–11].

The mapped reads were analyzed using several mutation-calling algorithms. Mutations with an AF of 0.1–0.8 were selected as candidate mutations. Mutations that were detected in two or more samples were eliminated to exclude potential false positive mutations. Mutations that were supported by fewer than 10 reads were also eliminated to enhance the reliability of our data. After removing mutations that were present in the parental lines, the remaining candidate mutations were confirmed using the IGV visualization software. A total of 769 mutations were detected in the seven independent anthocyanin-less sectors (Table 1). All detected mutations were categorized into five small-mutation groups, namely 1) single base substitution (SBS), 2) 1-bp insertion (+1 INS), 3) 1-bp deletion (−1 DEL), 4) insertion of 2–99 bp (2–99 INS), and 5) deletion of 2–99 bp (2–99 DEL), or three large-mutation groups, namely 6) insertions of 100 bp or more (≥100 INS), 7) deletions of 100 bp or more (≥100 DEL), and 8) structural variation (SV), or a single complex-mutation group, namely 9) complex. The complex group included mutations comprising two or more consecutive SBSs and mutations consisting of SBSs and small insertions/deletions (indels) separated by less than 10 bp; the origin of these mutations is presumably different from that of single SBS or deletion mutations [9–11]. None of the candidate mutations belonged to the ≥100 INS group. For each mutation group, 42 representative mutations with a broad AF (0.122–0.548) were selected and their sequences were verified by PCR and Sanger sequencing. All selected candidate mutations were confirmed as true mutations, which reflected the validity of this mutation detection method (S2 Table).

**Table 1:** Types of mutations detected in anthocyanin-less sectors.

Mutation type	Name of mutant sectors							Grand total
	5–3	8–5	13–2	15–4	22–3	27–4	29–4	
SBS	72	73	75	64	90	42	51	467
+1 INS	2	4	3	4	5	4	2	24
-1 DEL	10	14	8	15	15	15	5	82
2–99 INS	1	0	0	1	1	2	1	6
2–99 DEL	31	15	9	15	20	13	8	111
≥100 DEL	1	1	3	2	1	3	1	12
SV	0	1	3	1	4	4	0	13
complex	16	9	8	5	5	3	8	54
total	133	117	109	107	141	86	76	769

**Fig 2:**
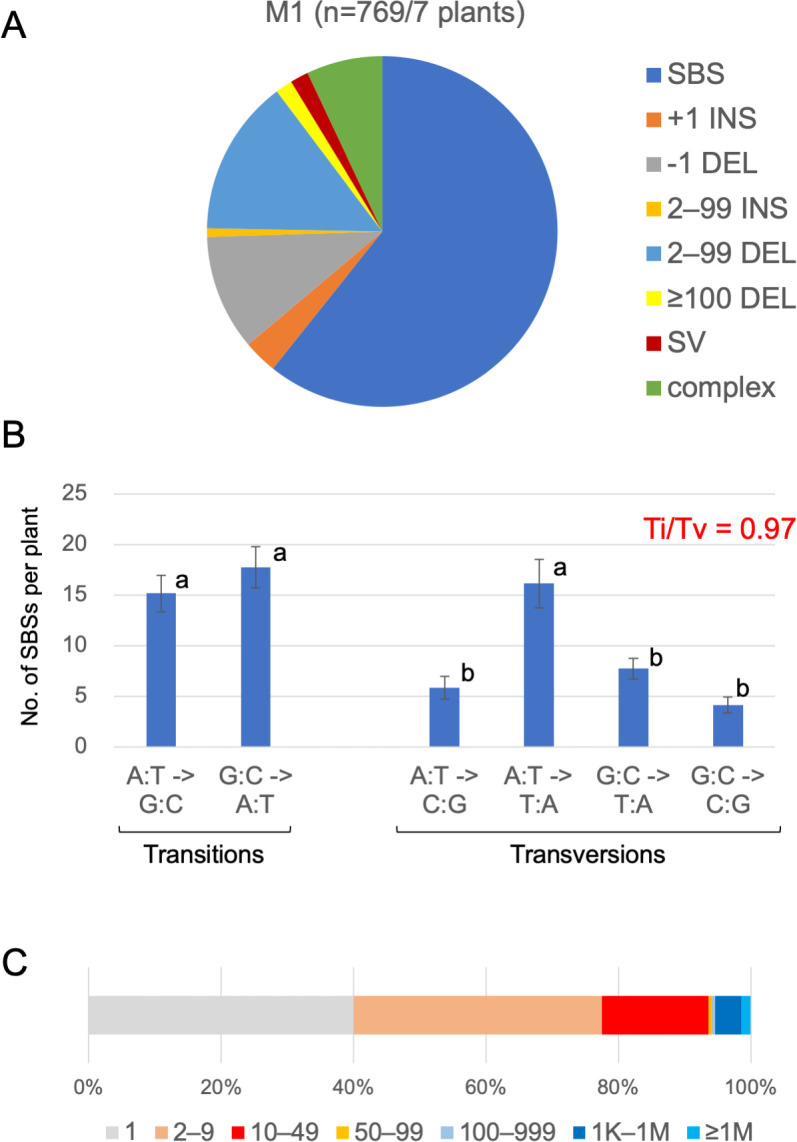
Mutation spectrum for the DNA in the examined sectors following the gamma-irradiation of seeds. (A) Spectrum of all mutations detected in seven anthocyanin-less sectors. (B) Characterization of SBS. Letters indicate statistical significance (one-way ANOVA with post hoc Tukey test, *p* < 0.05). The transition:transversion ratio (Ti:Tv) is indicated at the top right. (C) Distribution of deletion sizes. Sizes (bp) are indicated at the bottom.

**Fig 3:**
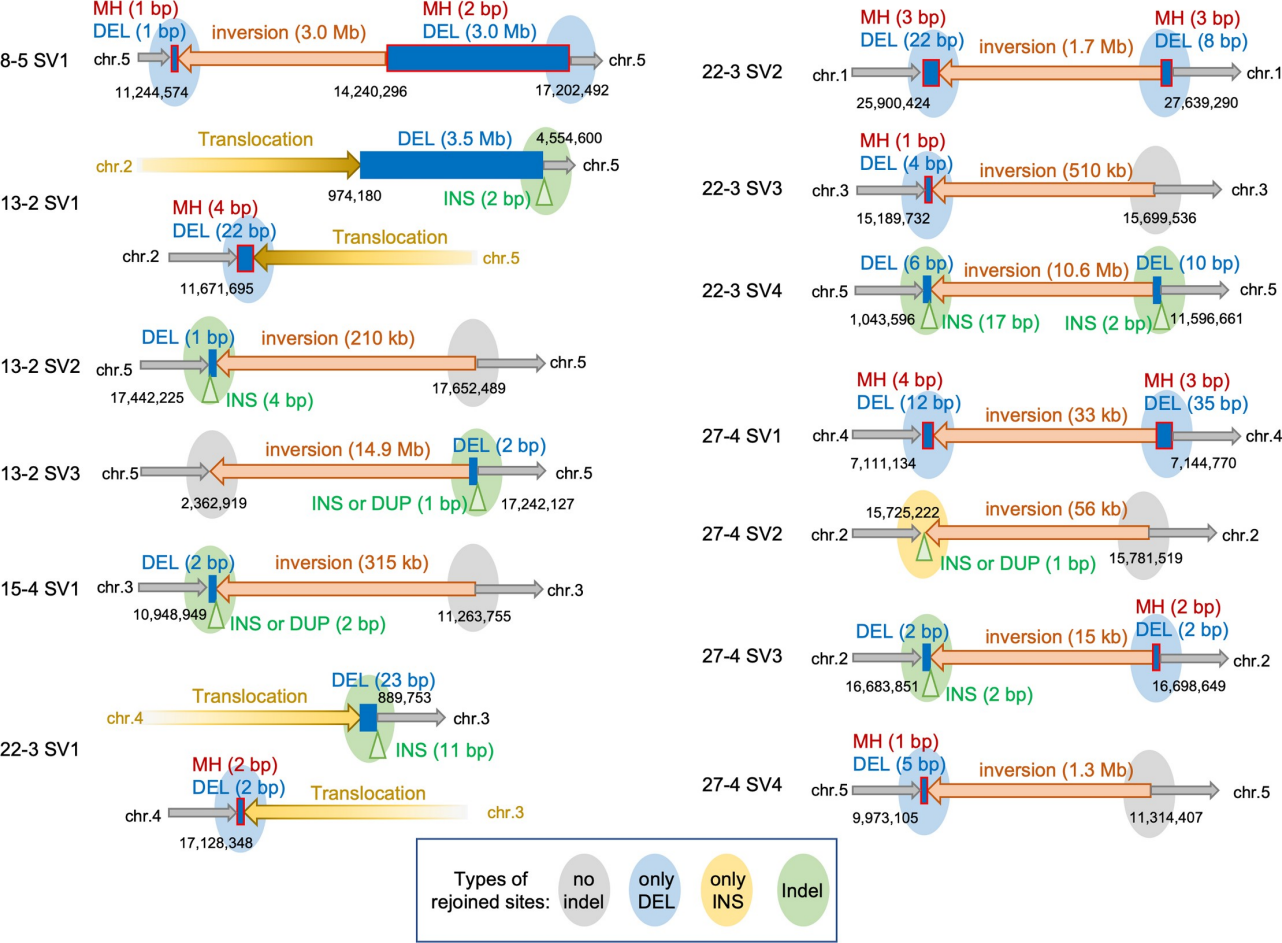
Schematic representation of all SVs detected in this study. Gray arrows indicate non-mutated genome sequences. Red and yellow arrows indicate inversions and translocations, respectively. Blue boxes indicate deletions and green triangles indicate insertions or potential duplications at the rejoined sites. Deletions with MH are indicated by blue boxes with a red outline. The types of rejoined sites are indicated by ovals with different colors: no-indel sites are gray, only-deletion sites are blue, only-insertion sites are yellow, and indel sites are green. Genomic coordinates are indicated as black letters. Abbreviations: DEL, deletion; INS, insertion; DUP, potential duplication.

SBSs were the most prominent mutation, representing 60.7% of all mutations (Fig 2A). More specifically, A:T to T:A, G:C to A:T, and A:T to G:C were the major substitutions (Fig 2B). The transition:transversion ratio (Ti:Tv) of 467 SBSs was 0.97 (Fig 2B). Insertions were minor mutations (about 4% of all mutations) and deletions accounted for about 25% of all mutations. The size distribution of the deletions in the M1 genome (n = 205) was determined (Fig 2C). Deletions of less than 10 bp represented about 75% of all deletions. Increases in the size of the deletions were generally associated with decreases in their frequency, although 12 of the mutations in the M1 genome belonged to the ≥100 DEL group, including mutations with a deletion of up to 5 Mb. No deletions were present in the centromere regions. Another 13 mutations belonged to the SV group, 11 of which were inversions, and the remaining two were reciprocal translocations. The length of the inversions detected in this study ranged from 15 kb to 14.9 Mb (Fig 3).

Mutations disrupting one of three wild-type *TT* genes at heterozygous loci were detected in five anthocyanin-less sectors, suggesting these mutations were responsible for the anthocyanin-less phenotypes. Mutations in *TT3* were found in three sectors, whereas mutations in *TT4* and *TT18* were detected in one sector each (Table 2). Of the five mutations in the *TT* genes, four belonged to the ≥100 DEL group, with deletions ranging from 519 kb to 5.0 Mb. The remaining mutation belonged to the −1 DEL group (Table 2). All mutations responsible for the anthocyanin-less phenotypes had an AF of approximately 0.3–0.4 (Table 2). There was no direct evidence of the cause of the anthocyanin-less phenotype in the two remaining sectors.

**Table 2:** Mutations responsible for the anthocyanin-less phenotypes.

Name of mutant sectors	Type, size, and locus of mutations	Allele frequency
5–3	not detected	
8–5	deletion (3.0 Mb) including TT3*	0.330
13–2	deletion (3.6 Mb) including TT4**	0.300
15–4	not detected	
22–3	deletion (519 kb) including *TT18*	0.342
27–4	deletion (5.0 Mb) including *TT3*	0.319
29–4	deletion (1 bp) in *TT3*	0.391

*, accompanied with 3.0 Mb inversion.

**, accompanied with reciprocal translocation.

### Characterization of SVs and large deletions

Because this mutation analysis of the M1 genome was able to reveal large DNA rearrangements (13 and 12 mutations in the SV and ≥100 DEL groups, respectively) that were not well-characterized in previous investigations of M2 genomes, we focused on characterizing the features of the SVs and large deletions.

In this study, 13 candidate SVs (11 inversions and 2 reciprocal translocations) were detected in the gamma-irradiated M1 genome. All of these mutations were confirmed by PCR and Sanger sequencing. The SVs identified in this study are presented in Fig 3, with sequence details provided in S3 Fig. Twenty-six junctions of the 13 SVs were divided into the following four types: 6 no indel sites, 11 only-deletion sites, 1 only-insertion site, and 8 indel sites (Fig 3). The deletions ranged from 1 bp to Mb-order, and deletions of up to 5 bp were overrepresented (9 of 19 deletions). The insertions ranged from 1 to 17 bp, and insertions of up to 5 bp were overrepresented (six of nine insertions). Thus, rejoined sites with 0–5 bp indels represented most of the SVs (16 of 26 rejoined sites; 61.5%, Fig 3). Notably, almost all of the insertions were located at the rejoined sites accompanied by deletions. Of the nine insertions, three small insertions (13–2_SV3 site 2 ‘G’, 15–4_SV1 site 1 ‘AC’, and 27–4_SV2 site 1 ‘A’) were potentially derived from duplications. Additionally, all only-deletion sites had MH at their junctions (blue boxes with a red outline in Fig 3). Among the SV junctions, we focused on the deletion-associated rejoined sites (11 only-deletion sites and 8 indel sites) to compare deletion characteristics.

**Fig 4:**
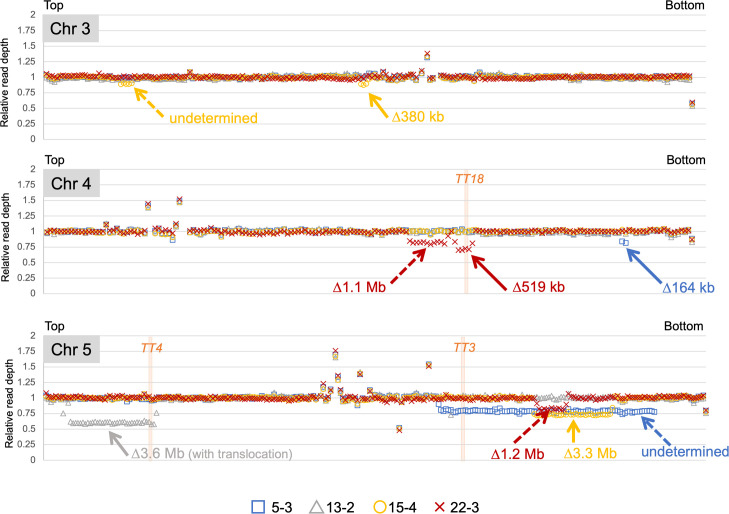
Distribution of NGS reads to the reference sequences (100 kb-bin size) on chromosome 3, 4 and 5 for four M1 mutant sectors [5–3 (blue squares), 13–2 (gray triangles), 15–4 (golden circles) and 22–3 (red crosses)]. Large deletions are labeled by individual mutation names. In addition to the large deletions found by the mutation-calling algorithms (indicated by solid arrows), four additional regions (dotted arrows) showed decrease of the reads. For the regions of chromosome 5 in 5–3 (blue dotted arrow) and chromosome 3 in 15–4 (golden dotted arrow), probable junctions could not be detected from our NGS data (labeled as “undetermined”). Approximate positions of three heterozygous *TT* genes are labeled with pale orange bars. Distribution of NGS reads on all chromosomes for all 7 M1 mutant sectors are shown in S4 Fig.

To compare the characteristics of the deletion-associated rejoined sites of SVs, the rejoined sites of the ≥100 DEL mutations were analyzed because the SV and ≥100 DEL mutations are thought to be derived from the incorrect rejoining of two distally located DSBs. As mentioned above, the mutation-calling algorithms used here detected 12 candidate mutations as the ≥100 DEL group. A different approach, a dosage analysis of read coverage in chromosomal bins [25], was also conducted for detecting large chromosomal segments having more or less dosage than other chromosomal regions. In this analysis, relative read depth (RRD) was calculated by dividing the read depth in non-overlapping 100 kb-bins with the median depth of coverage for 5 chromosomes in each line. When the RRD in each bin was plotted to the corresponding chromosomal region, most of chromosomal regions are expected to show RRD as around 1, whereas deleted regions of several hundreds kb in size would be realized as consecutive bins of 100-kb with reduced RRD values that depend on the extent of the chimerism. As like in the mutation detection method where minimum AF of candidate mutations was 0.1, chromosomal regions showing RRD of <0.9 were selected as candidates for large deletion in dosage analysis. This analysis did not detect whole chromosome aneuploidy, but identified 12 chromosomal regions in which the averaged RRDs were below 0.9 (S4 Fig, arrows). Eight out of the 12 regions were also detected by the mutation detection method described above as the ≥100 DEL candidates, whereas the remaining four were new candidates (Fig 4). Examining the reads at the boundary of each putative deletion by using IGV, detected possible junctions for the two new ≥100 DEL candidates but not the other two. We also found at least 3 chromosomal regions in which the averaged RRDs were slightly decreased but not less than 0.9 (S4 Fig, arrowheads). The reads located at the putative boundary of these three potential deletions could not also be found by IGV, presumably due to insufficient number of reads for detecting such a low population of these potential deletions in the sector. Therefore, except for the potential deletion candidates whose junctions were unclear, a total of 14 ≥100 DEL candidates including two candidates identified by dosage analysis, were analyzed in later characterization.

**Fig 5:**
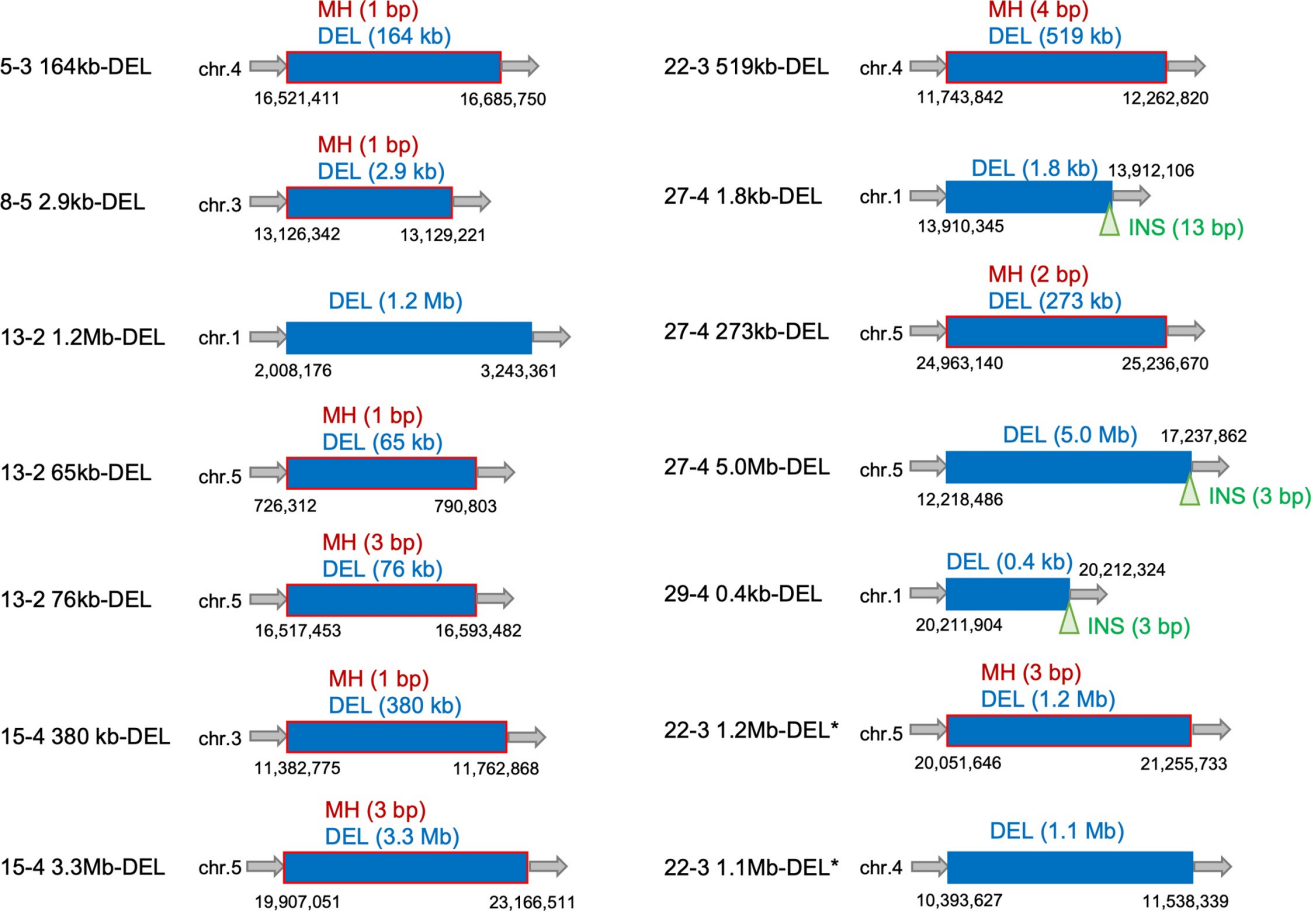
Schematic representation of all large deletions (≥100 bp) detected in this study. Gray arrows indicate non-mutated genome sequences. Blue boxes and green triangles indicate deletions and insertions at rejoined sites, respectively. Deletions with MH are indicated by blue boxes with a red outline. Asterisks indicate two large deletions found by dosage analysis. Genomic coordinates are indicated as black letters. Abbreviations: DEL, deletion; INS, insertion; MH, microhomology.

Similar to the analysis of SVs, a total of 14 candidate mutations in the ≥100 DEL group were analyzed by PCR and Sanger sequencing, which confirmed that all of the candidates were true large deletions. The size of the deletions varied from 0.4 kb to 5.0 Mb (Fig 5). Three of these 14 mutations had insertions at their rejoined sites (21.4%) (Fig 5). The insertions were 3, 3, and 13 bp long. Moreover, 9 of the 14 mutations had MH at the junctions (64.3%). These results indicate that the features and the frequency of insertions and the MH frequency in the ≥100 DEL group were similar to those in the SV group. This is consistent with the fact large deletions (≥100 bp) were grouped with SVs and categorized as rearrangements in earlier studies (e.g., [8,26]). Therefore, the rejoined sites of the deletion-associated SV and the ≥100 DEL mutations were merged and designated as rejoined sites of rearrangements.

### Comparison of the characteristics of rearrangements with small and medium deletions

**Fig 6:**
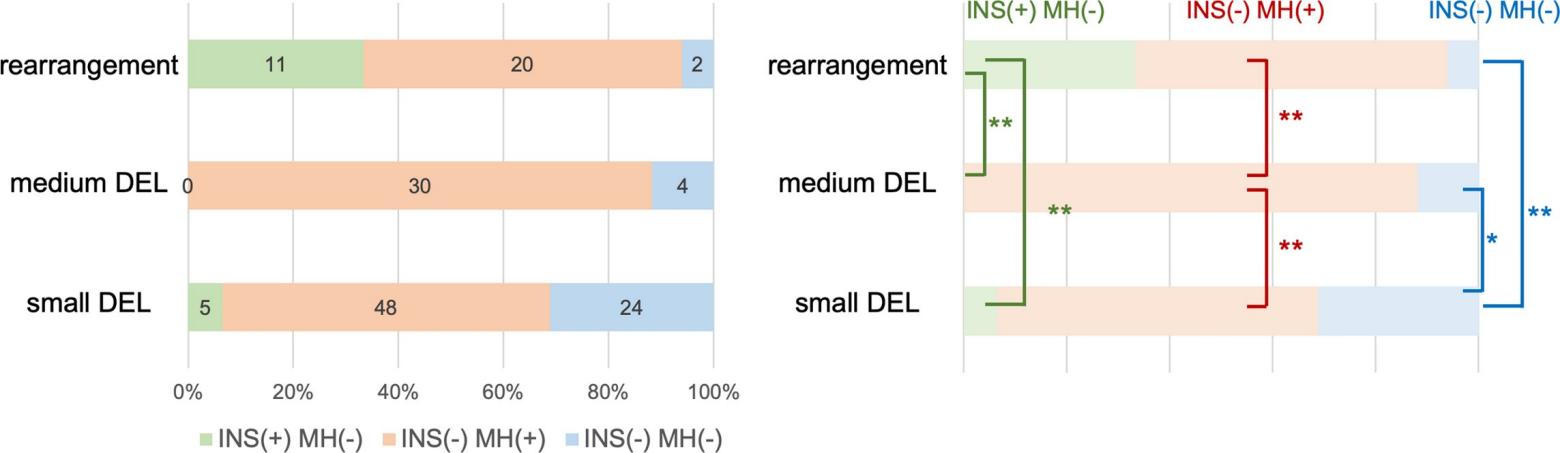
Comparison of the characteristics among rearrangements, medium DELs, and small DELs. The ratio and number of rejoined sites with an insertion and without MH [INS(+) MH(−)], without an insertion and with MH [INS(−) MH(+)], and without an insertion or MH [INS(−) MH(−)] are provided in the left panel. Values in the colored boxes represent the number of detected rejoined sites. Significant differences in each category are indicated in the right panel. * *p* < 0.05, ** *p* < 0.01 (Fisher’s exact test). Abbreviations: DEL, deletion; INS, insertion; MH, microhomology.

To further characterize the rearrangements mentioned above, the rejoined sites of rearrangements were compared with the 2–99 DEL mutations, most of which formed via DSB repair. The 2–99 DEL mutations were classified as small DELs (2–9 bp) and medium DELs (10–99 bp) (Fig 6). The frequency of insertions at the rejoined sites was significantly lower for both small DELs (5 of 77; 6.5%) and medium DELs (0 of 34; 0%) than for the rearrangements (11 of 33; 33.3%, *p* < 0.01, Fisher’s exact test) (Fig 6). Next, the presence of MH at the rejoined sites was investigated. The frequency of MH in the rearrangements was 60.6% (20 of 33), which was similar to that in the small DELs (48 of 77; 62.3%). In contrast, the frequency of MH in the medium DELs was 88.2% (30 of 34), which was significantly higher than the corresponding frequencies in the small DELs and the rearrangements (*p* < 0.01, Fisher’s exact test) (Fig 6). The potential relationship between deletion size and MH length was also investigated. Because the deletions with 2-bp MH are reportedly the most common type in the ≥2 DEL group in the M2 generation of gamma-irradiated wild-type Arabidopsis [10], the threshold for MH length was set between 2 bp (≤2 bp) and 3 bp (≥3 bp) for each of the three categories. The proportion of ≥3 bp MH was highest in the medium DELs (57%), followed by the rearrangements (45%) and the small DELs (25%) (S5 Fig). Finally, the frequency of rejoined sites with no insertion or MH was determined. Rejoined sites that lacked an insertion and MH were significantly more common in the small DELs (24 of 77 sites; 31.2%) than in the medium DELs (4 of 34 sites; 11.8%) and the rearrangements (2 of 33 sites; 6.1%) (Fig 6). These results indicate that rearrangements as well as medium and small DELs have distinct features at their rejoined sites. This suggests different processes preferentially formed each of these mutations.

**Fig 7:**
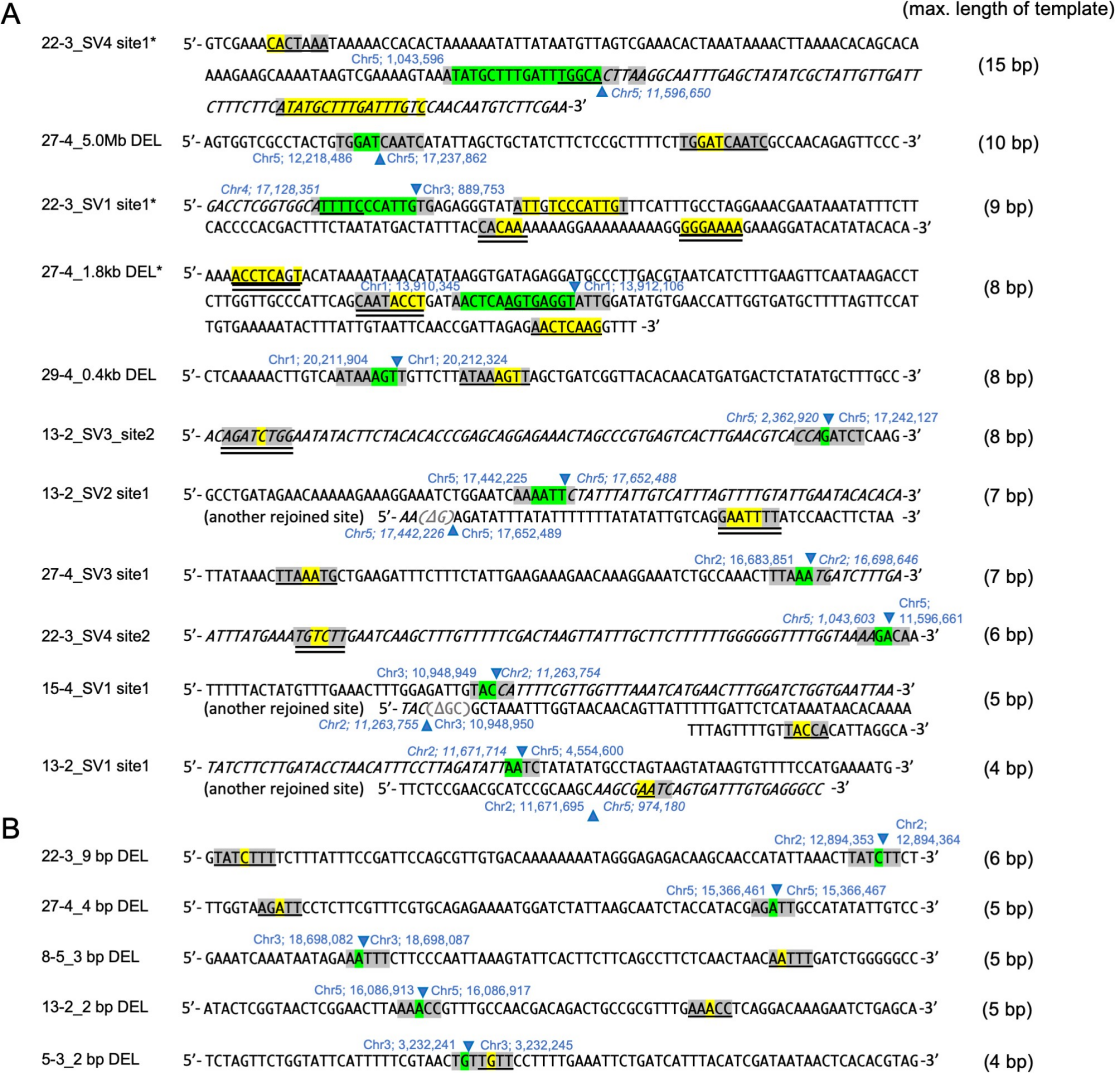
Sequences around the junctions harboring insertions in the rearrangements (A) and small DELs (B). The longest template sequences are indicated on the right side. Inserted nucleotides are highlighted in green. Candidate templates for the insertions are labeled with a single underline (direct strand) and a double underline (reverse complement). Homologous nucleotide sequences between the template and the insertion are highlighted in yellow. Homologous nucleotide sequences between the sequences flanking the template and the insertions are highlighted in gray. Three junctions harboring insertions of ≥10 bp are labeled with asterisks. Broken and/or rejoined sites are indicated with blue triangles. Genomic coordinates are indicated at both sides of the inserted sequences. Deleted nucleotides are indicated as gray letters. Rejoined sequences with inverted direction by SVs are indicated as italic.

Because the high frequency of insertions was a unique feature of rearrangements, the inserted sequences were further analyzed to determine their origins. We searched for sequences that were homologous to the insertions and their flanking regions within 100 bp of the junctions, and found their potential template sequences near one of the junctions (Fig 7A). These template sequences were relatively long, varying from 4 to 15 bp. Most of the template sequences were identical to the insertions and their flanking regions, but for three large insertions (marked with asterisks in Fig 7A), multiple stretches of sequences that were highly homologous to the insertions and their flanking regions were found; these templates were up to 15, 9, and 8 bp long at each junction (Fig 7A). Such a relatively long template was not detected in the junctions of the small DEL mutations, in which all insertions were 1 bp long and their potential templates were 4 to 6 bp long (Fig 7B).

### Evaluation of mutations that occurred in a single ancestral cell of the M1 sector

**Fig 8:**
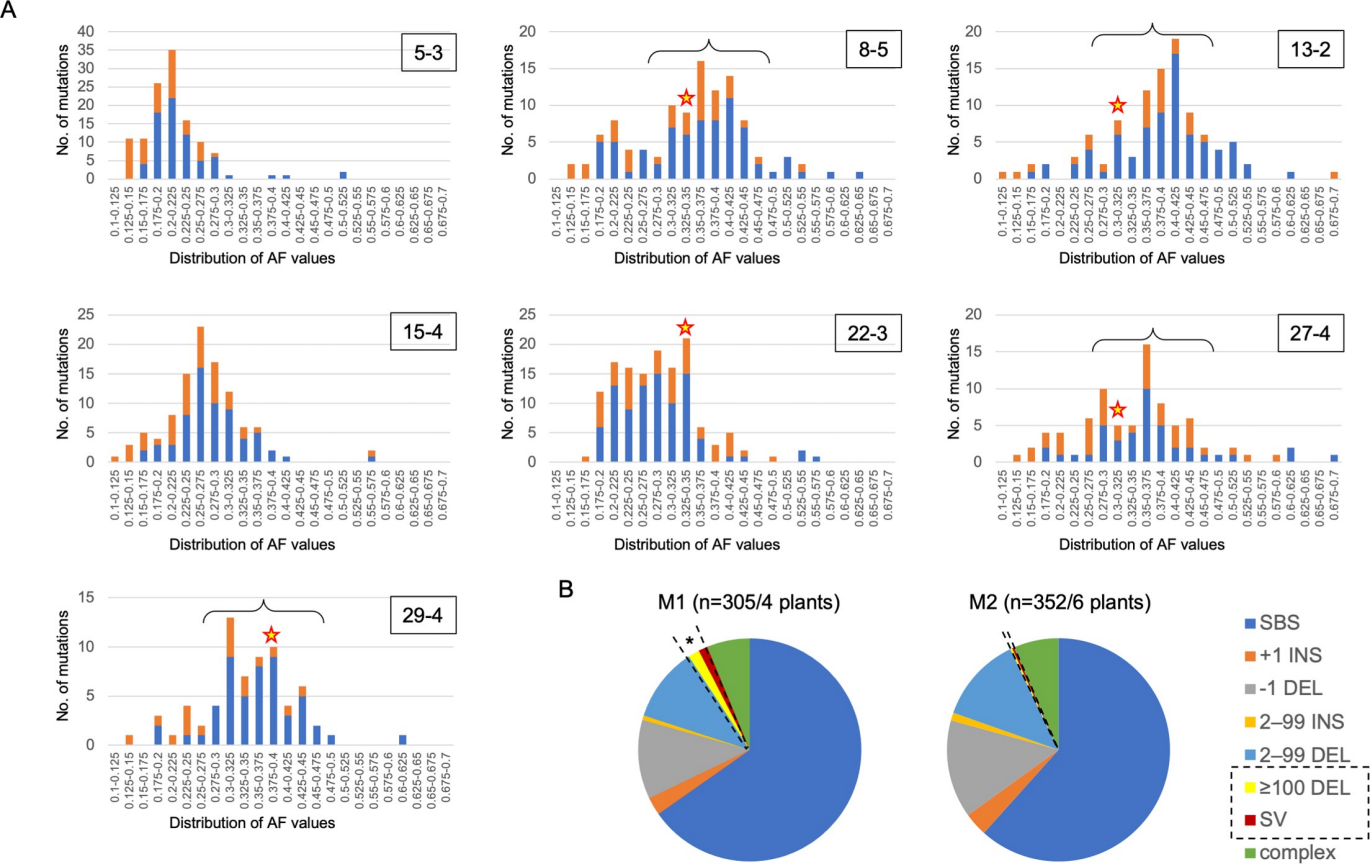
Characterization of mutations on the basis of the AF. (A) Distribution of AFs of mutations [indels (orange) and SBSs (blue)] in anthocyanin-less sectors. Data were analyzed using GATK. Stars indicate the AF of the mutations responsible for the anthocyanin-less phenotypes. The mutations indicated by the black curly brackets are from the major mutation groups and are used as the representative mutations that occurred in a putative single ancestral cell in a dry seed. (B) Mutation spectrum for the representative mutations in four M1 plants (8–5, 13–2, 27–4, and 29–4) (left) and in six M2 plants analyzed in a previous study (right; [11]). The proportions of the rearrangements are indicated by dotted lines. * *p* < 0.05 (Fisher’s exact test).

The anthocyanin-less cells likely originated from a single cell in the embryo that was irradiated at the seed stage and were expected to harbor an identical set of mutations. However, because the anthocyanin-less sectors were identified by eye and excised together with the abaxial anthocyanin-positive tissues, an unknown number of cells harboring a different set of mutations were included among the cells obtained from individual excised sectors. Anthocyanins in Arabidopsis are accumulated in subepidermal cells on the adaxial side of leaves and in epidermal cells on the abaxial side of leaves [27]. Accordingly, the anthocyanin-less cells we screened in this study were likely subepidermal cells. The subepidermal cells in Arabidopsis originate from cells in the L2 layers, similar to the main leaf tissue parts, including the palisade mesophyll and the outer spongy mesophyll [28–30]. Therefore, the anthocyanin-less cells harboring an identical set of mutations were expected to be abundant in sectors on mature Arabidopsis leaves. Because such an abundance of cells harboring the identical set of mutations in the sectors was considered to reflect the AF of each mutation in the NGS data, the AFs of the identical set of mutations including *tt* mutation were expected to be relatively high but variable depending on the extent of existing rate of other cells harboring a different set of mutations in the sectors. To confirm this, the distribution of mutations along with the AFs were investigated for each sector. Regarding sectors 5–3 and 15–4, in which we failed to detect the mutations responsible for the anthocyanin-less phenotypes, the AF peaks were around 0.2 and 0.25, respectively (Fig 8A). In addition, as shown in Fig 4, a chromosomal region harboring wild type *TT3* gene in the sector 5–3 was detected as low dosage region in which relative read depth was about 0.75–0.8, suggesting that rate of the anthocyanin-less L2 cells was about 0.2–0.25 in the sector 5–3. One possible explanation of these low AF peaks is that growth of L2 cells harboring *tt* mutation was inhibited by coexisting deleterious mutations, resulting in lower frequency of the anthocyanin-less L2 cells than expected in these sectors. An analysis of sector 22–3 revealed the mutations were scattered, with AFs of 0.2 to 0.35, presumably because two peaks were close to each other (Fig 8A). For the remaining sectors (8–5, 13–2, 27–4, and 29–4), the peak AF was around 0.35 or higher (Fig 8A), indicating that most of the mutations were common among the excised sectors. Additionally, the AFs of the mutations responsible for the anthocyanin-less phenotypes were mapped in the major peaks in all four sectors (Fig 8A). These results are consistent with the prediction that the anthocyanin-less cells harboring an identical set of mutations represent the major parts of the anthocyanin-less sectors. Moreover, these mutations were likely derived from a single ancestral cell. Therefore, using the mutations in the major peak (AF of 0.25 to 0.5) in these four sectors as representatives, the mutation frequency and spectrum were evaluated by assuming the mutations occurred in a single M1 cell in dry seeds after the irradiation. The mutation frequencies obtained by this approach ranged from 5.37 × 10^−7^ to 7.30 × 10^−7^ per base pair in each of the four sectors (average of 6.40 × 10^−7^ per base pair), which is similar to the predicted mutation frequency in an earlier mutation analysis of the M2 genome (average of 6.55 × 10^−7^ per base pair; [11]) (Fig 8B). Regarding the mutation spectrum, the overall patterns in the M1 genome were likely similar to those in the M2 genome. However, the proportion of the rearrangements was significantly higher in the M1 genome than in the M2 genome (*p* < 0.05, Fisher’s exact test) (Fig 8B).

## Discussion

### Mutations occurred in the SAM of dry seeds

The genome-wide effects of mutagens may be more clearly revealed by exploring the M1 genome sequence rather than the genomes of M2 or later generations because the M1 genome is directly irradiated. Moreover, examining the M1 genome avoids the potential loss of mutations that may occur during gametogenesis. Additionally, for several ornamental species that can be propagated via vegetative clonal methods, the mutational characterization of the M1 generation may provide valuable information related to their genetic improvement. However, detecting mutations throughout the M1 genome is difficult because of chimerism; many types of mutated cells are present in varying proportions. One strategy for accurately detecting infrequent mutations involves sequencing specific loci with an extremely high coverage depth (e.g., >100,000× in Narayan et al., 2012 [31]). Unfortunately, WGS at such a coverage depth is impractical for plants. For example, the Arabidopsis genome comprises >10^8^ bp, which is considerably greater than the <50 bp DNA region analyzed by Narayan et al. (2012) [31]. In the present study, we employed a different approach, which involved the mutagenesis of genetically heterozygous seeds and the subsequent detection of mutations on the basis of LOH after the mutated clonal cells were grown. This made it possible to identify tissues comprising only a few mutated ancestral cells. The WGS analysis of these materials resulted in a highly reliable overview of the genome-wide mutations in the M1 generation (Fig 2 and Table 1). Using this experimental approach, we were able to reveal for the first time the mutation profile of an M1 plant genome.

An in-depth characterization of the AFs for all mutations enabled the identification of the major mutation groups, including the radiation-induced *tt* mutations in four sectors (Fig 8A). An anthocyanin-less sector was the result of the division of single cells harboring mutations at any of the three heterozygous *TT* loci. We focused our analysis on the major mutation groups in the four sectors because these mutations were probably induced in a single ancestral cell in the SAM of dry seeds exposed to gamma-rays. The anthocyanin-less sectors were detected specifically on the adaxial side of leaves (Fig 1B). A previous study indicated that anthocyanins accumulate in subepidermal cells on the adaxial side of leaves [27]. Accordingly, we speculated that the mutations, including the *tt* mutation, occurred in an ancestral cell of the L2 layer in Arabidopsis seeds. It is generally accepted that L2 cells contribute to the generation of gametophytes (germlines) [32,33]. In Arabidopsis, a subset of cells, including germlines, undergo a limited number of divisions and protect their genomes from spontaneous mutations more strictly than other SAM cells [34,35]. These results suggest that functional germlines are segregated during the early stage of development, and are inconsistent with a previously proposed theory that plants do not define germlines early in development [12,36,37]. From this viewpoint, it is important to show that the gamma-irradiation-induced mutations in M1 vegetative cells are similar to those previously detected in M2 cells (derived from germline mutations in M1 [11]) (Fig 8B). Although it is unclear whether the functional germlines are defined in the seed stage, our results imply that there are no major differences in the occurrence of radiation-induced mutations in SAM cells in seeds, irrespective of whether they will form vegetative or reproductive cells. However, the proportion of the rearrangements was significantly higher in the M1 genome than in the M2 genome (Fig 8B). Seven out of the nine rearrangements in single M1 cells were involved in large deletions (including ones occurred accompanied with SVs) (see S3 Table, the nine rearrangements are marked by **). Together with no ≥100 INS mutation in M1 genomes, a net loss of the four gamma-irradiated Arabidopsis M1 genomes was estimated of about 13 Mb (3.2 Mb per M1 genome). Such a great loss of DNA by gamma-irradiation is consistent with a hypothesis for ongoing shrinking of the Arabidopsis genome via deletion-biased DNA repair [38], and the cells harboring those large deletions are likely not involved in the formation of viable gametes or seeds [39].

### Molecular nature of the mutations induced by gamma-rays

There are several reports describing genome-wide mutations induced by gamma-rays in M2 or later generations in plants [11,26,40]. Regarding small mutations, specific characteristics, such as the SBS spectrum and the Ti:Tv ratio in M1 (Fig 2B and Table 1), were similar to previously observed characteristics in M2 or later generations. In contrast, large DNA modifications, including rearrangements, have rarely been detected in M2 or later generations. For example, earlier investigations uncovered only one ≥100 DEL mutation in three M2 tomato plants [40], one SV mutation in seven M5 rice plants [26], and one SV mutation and one ≥100 DEL mutation in six M2 Arabidopsis plants [11]. In the current study, 27 rearrangements (13 SV and 14 ≥100 DEL mutations) were detected in seven M1 Arabidopsis plants. Considered together, these findings clarified the differences in the sensitivity of detecting mutations in the M1 and M2 genomes, especially large DNA alterations. These results also demonstrated that analyzing the M1 genome for mutations is conducive to detecting and characterizing large DNA alterations. Very recently, Guo et al. (2021) [41] conducted WGS analysis on F1 poplar plants obtained with gamma-irradiated pollen. Although the authors focused on specific chromosomes with highly complex segmental rearrangements formed by “chromoanagenesis [42]”, they detected a number of Mb-ordered deletions and duplications, implying that their system, similar to our own, was well suited for detecting large DNA alterations.

Several studies using artificially inducible DSB systems support the idea that DSBs are predominantly repaired via c-NHEJ in Arabidopsis [43–45]. However, the mechanisms underlying the repair of DSBs induced by gamma-irradiation remain relatively unknown, although there are reports of the hypersensitivity of c-NHEJ-depleted Arabidopsis mutants to ionizing radiation [10,46,47]. Because rearrangements occur during the repair of DSBs, the successful characterization of 27 rearrangements in the gamma-irradiated M1 Arabidopsis genome (Figs 3, 4 and 5) generated valuable information that was lacking in the previous investigations of M2 genomes. First, we detected 9 insertions and 19 deletions at the junctions of SVs, most of which were up to 5 bp (Fig 3). The six no-indel sites combined with the rejoined sites with indels less than 5 bp represented the majority of SVs (61.5%). In the ≥100 DEL group, short insertions (<5 bp) were also the predominant mutations (66.7%). Because c-NHEJ generates no indels or very short indels at the rejoined sites [48,49], the junction patterns in the rearrangements suggest that c-NHEJ is crucial for repairing gamma-irradiation-induced DSBs, which is consistent with the fact that DSBs in Arabidopsis are primarily repaired by the c-NHEJ pathway.

Another characteristic of rearrangements, which are derived from the incorrect rejoining of two distal DSB ends, is the high frequency of insertions at their junctions (Fig 6). A detailed junction analysis detected template sequences for the insertions and the flanking regions near the rejoined sites, but their lengths differed between the rearrangements and small DELs. Although the template sequences were 4–6 bp in the small DELs, the template sequences for the rearrangements were up to 15 bp long (Fig 7). Polymerase θ (Pol θ) generates insertions at the junctions of rearrangements in mammals [50]. The presence of long template sequences and sequences identical to the sequence flanking insertions are signatures of Pol θ-mediated DSB repair [44,51]. In Arabidopsis, although there is no direct evidence of Pol θ generating insertions, similar signatures of Pol θ-mediated DSB repair have been observed at T-DNA integration sites [52] and at the rejoined sites of CRISPR/Cas9-induced DSBs [17,44]. Therefore, the long template-derived insertions detected in gamma-irradiation-induced rearrangements may be formed via the Pol θ-mediated repair pathway. In other words, Pol θ may contribute substantially more to the rejoining of two distal DSB ends and the formation of rearrangements than to the rejoining of proximal DSB ends and the formation of small DELs in gamma-irradiated plant genomes.

In addition to the features of rearrangements described above, there were some other interesting findings regarding the small and medium DELs. Specifically, the MH frequency was much higher for medium DELs than for rearrangements or small DELs (Fig 6). This implies that the formation of medium DELs is associated with MH. In mammals, MH-mediated end-joining (MMEJ) is an MH-dependent DSB repair pathway, in which DSBs are processed by nucleases to generate MH at both ends, which are subsequently rejoined [53]. Additionally, MMEJ is sometimes used as a part of or instead of alt-EJ, and it seems to function under c-NHEJ-depleted conditions [50]. Therefore, our data indicate that medium DELs are preferentially formed by an MH-dependent pathway in gamma-irradiated Arabidopsis. The process is likely similar to MMEJ in mammals. At the rejoined sites of enzymatically-induced DSBs in Arabidopsis, deletions shorter than 10 bp are reportedly the predominant mutation in wild-type plants, but not in *atku70* mutants in which 10–49 bp deletions are more common [43]. Similarly, in a recent study, 11–30 bp deletions were detected more frequently in the gamma-irradiated M2 genome of *atku70* than 2–10 bp deletions, which were the predominant mutations in wild-type plants [10]. Because *atku70* is a c-NHEJ-depleted mutant, medium-sized deletions [10–49 bp in Qi et al. (2013) [43] and 11–30 bp in Du et al. (2020) [10]] are likely generated in the absence of the c-NHEJ pathway in Arabidopsis. Although a relationship between deletion size and MH dependence has not been analyzed, the predominance of medium-sized deletions in c-NHEJ-depleted mutants is consistent with our hypothesis that the formation of medium DELs depends on an MMEJ-like mechanism.

The current study also revealed that the frequency of rejoined sites with neither insertions nor MH was higher for small DELs than for rearrangements and medium DELs (Fig 6). If the MH frequency reflects, at least partly, the extent of the processing of DSBs by nucleases, our result indicates that small DELs are mainly formed by the rejoining of DSBs with minimal processing (e.g., c-NHEJ). The decreased frequency of deletions shorter than 10 bp in the irradiated genome of *atku70* Arabidopsis mutants [10] also indicates that these small deletions (<10 bp) are formed mainly in a Ku-dependent manner, probably via c-NHEJ, which supports our hypothesis. Using our system to conduct additional genome-wide mutational analyses of materials with diverse genetic backgrounds, including materials lacking one of the factors mediating DSB repair pathways, will promote our understanding of the formation of various mutations, including rearrangements, in plants.

In conclusion, genome-wide analyses of mutations induced by gamma-rays were conducted by isolating M1 tissues with minimal chimerism using a triple heterozygous Arabidopsis line. Our strategy facilitated the comparison of mutation frequencies and spectra between M1 and M2 genomes. The mutations in the two genomes were similar, but the proportion of rearrangements was higher in the M1 genome. We determined that the rejoined sites of gamma-irradiation-induced SVs can be classified as no-indel sites, only-deletion sites, only-insertion sites, and indel sites. An examination of junction sequences revealed that rearrangements as well as medium and small DELs had distinct features at their rejoined sites (i.e., the frequency of insertions and MH). Accordingly, the rearrangements, medium DELs, and small DELs are formed preferentially via different processes. Furthermore, for some insertions in rearrangements, there were sequences highly homologous to the insertion and the flanking region near the junctions, implying they were formed by Pol θ activity. Our study provides valuable information related to the nature of genome-wide mutations induced by gamma-irradiation. Moreover, the system used in this study is applicable for investigating the effects of mutagens on the M1 genome of plant species with diverse genetic backgrounds.

## Materials and methods

### Plant materials

Arabidopsis flavonoid-less mutants *transparent testa4* (*tt4*), *tt3*, and *tt18* were used in this study. A triple mutant harboring the transposon (d*Spm*)-inserted *tt4* allele (line CS103730; [54]), the ion-beam-induced *tt3* allele (*tt3-3*; [4]), and the T-DNA-inserted *tt18* allele (SALK_073183; [55]) was obtained via cross pollination. Three additional generations of the triple mutant were propagated by single-seed descent. Wild-type Columbia seeds were also obtained using a similar single-seed propagation method. The single-seed-propagated triple mutants were crossed with the wild-type control, and the F1 hybrid seeds containing three heterozygous *tt* genes (*TT4tt4 TT3tt3 TT18tt18*) were obtained. Dry triple heterozygous seeds were irradiated with ^60^Co gamma-rays (1,000 Gy) for 50 min at the Takasaki Advanced Radiation Research Institute, Japan. The irradiated seeds were immediately sown on a soil mixture comprising Metro-Mix 350 (Scotts-Sierra Horticultural Products, Marysville, OH), vermiculite (Hakugen, Tokyo, Japan), and TM-2 (Takii Seed, Kyoto, Japan) (1:1:1) and then incubated in a growth chamber set at 23°C with a 12-h light:12-h dark cycle. Growing M1 seedlings were analyzed, especially regarding the accumulation of anthocyanins. Anthocyanin-less sectors were excised from normal anthocyanin-positive tissues, frozen in liquid nitrogen, and then stored at –80°C unless they were used immediately.

### Mutation detection by next-generation sequencing

Total genomic DNA was extracted from anthocyanin-less sectors using the DNeasy Plant Mini Kit (Qiagen, Hilden, Germany). The genomic DNA was then used to construct NGS libraries using the KAPA HyperPlus Library Preparation Kit (Kapa Biosystems, Wilmington, MA) along with the IDT for Illumina unique dual indexes (Illumina, San Diego, CA). Sequencing libraries were also prepared for the parental lines (triple mutant and wild-type). The libraries were pooled at an appropriate concentration for the Illumina HiSeqX and NovaSeq6000 NGS with PE150 chemistry. Low-quality reads and adapter sequences were removed from the raw data using Illumiprocessor (version 2.0.9; https://illumiprocessor.readthedocs.io/en/latest/). The remaining clean reads were mapped to the Arabidopsis reference genome (TAIR10.27; https://www.arabidopsis.org/) using the Burrows–Wheeler Aligner (version 0.7.5; http://bio-bwa.sourceforge.net/), SAMtools (version 1.3.1; http://samtools.sourceforge.net/), and Picard-tools (version 1.119; https://broadinstitute.github.io/picard/). The obtained BAM files and the GATK HaplotypeCaller algorithm (version 3.4; https://gatk.broadinstitute.org/hc/en-us) were used to call mutations, including SBSs and indels. Large DNA alterations, such as inversions, translocations, duplications, and 10-bp or larger indels, were detected using BreakDancer (version 1.4.5; http://breakdancer.sourceforge.net/) and Pindel (version 0.2.4; http://gmt.genome.wustl.edu/packages/pindel/). Mutations with an AF of 0.1–0.8 in one line and an AF <0.05 in the other lines were designated as candidate mutations. Mutations called on the basis of fewer than 10 reads were considered to be unreliable. Polymorphisms in the parents, which were identified by comparing the NGS data for the parents, were eliminated. All candidate mutations were confirmed using IGV (version 2.9.4; https://software.broadinstitute.org/software/igv/). The mutations identified in this study were classified into eight groups (Table 1). The SV junctions included three 1–2 bp insertions, which were complementary to the nucleotides on the opposite side of the inverted fragment. Because they were small, these sequences were considered as insertions rather than potential duplications.

Dosage analysis [25] was conducted using Python script “bin-by-sam_v7.py” (https://github.com/Comai-Lab/bin-by-sam), with sam (sequence alignment map) files as input and 100 kb as the bin size. For each line, reads in NGS data were pooled into non-overlapping 100 kb-bins of the reference genome. The read depth in each bin was normalized by dividing with a median read depth for all 5 chromosomes in each line, obtaining relative read depth in each bin. The relative read depth in each bin was plotted to the corresponding chromosomal region.

### Validation of the candidate mutations

Target-specific PCR and Sanger sequencing of the PCR products were performed for the 44 candidate mutations (57 mutated sites), which were distributed in eight mutation categories (S2 Table). The PCR to amplify the target regions was completed using ≥0.1 ng DNA extracted from the sectors as the template along with AmpliTaq Gold 360 (Thermo Fisher Scientific, Waltham, MA) and target-specific primers (S2 Table). The PCR products were purified using the QIAquick PCR Purification Kit (Qiagen) and further purified using the QIAquick Gel Extraction Kit (Qiagen) if necessary. The rejoined sites of the rearrangements, such as the SVs and ≥100 bp DELs, were confirmed by directly sequencing the purified PCR products. Regarding the other types of mutations, especially those with an AF less than 0.3, it was sometimes difficult to determine the candidate sequences by direct sequencing because of the chimerism with non-mutated parental sequences. Thus, the PCR products were first cloned into pGEM-T Easy vectors (Promega, Madison, WI), after which 10–30 clones were randomly selected for a cleaved amplified polymorphic sequence (CAPS) analysis [56], which can discriminate the target mutated sequence from the parental sequence. The mutation-positive clones revealed by the CAPS analysis were subjected to Sanger sequencing to verify the mutations.

## Supporting information

S1 Fig Arabidopsis anthocyanin biosynthetic pathway. Anthocyanins are synthesized through sequential enzyme reactions involving chalcone synthase (CHS), chalcone isomerase (CHI), flavanone 3-hydroxylase (F3H), flavonoid 3’ hydroxylase (F3’H), dihydroflavonol 4-reductase (DFR), leucoanthocyanidin dioxygenase (LDOX), and UDP-glucose:flavonoid glucosyltransferase (UFGT). These enzymes are encoded by *TRANSPARENT TESTA4* (*TT4*), *TT5*, *TT6*, *TT7*, *TT3*, *TT18*, and *ANTHOCYANINLESS1* (*ANL1*), respectively. Three genes used in this study were underlined in right side of the pathway.
(TIF)

S2 Fig Phenotypes of 7 M1 plants harboring anthocyanin-less sectors. Red arrows indicate anthocyanin-less sectors analyzed by NGS. All plants were grown in 100 mm^2^ pots. Two plants (22–3 and 29–4) are also shown in Fig 1.
(TIF)

S3 Fig Junction sequences of SVs. Red bold letters indicate microhomologous sequences. Green letters indicate inserted sequences. Underlined bold letters indicate potentially duplicated sequences. Abbreviations: DEL, deletion; INS, insertion; INV, inversion.
(XLSX)

S4 Fig Distribution of NGS reads to five chromosomes of the reference sequences (100 kb-bin size). Eight ≥100 DEL candidates identified by the mutation-calling algorithms are indicated by solid arrows. Four additional ≥100 DEL candidates newly detected by this dosage analysis are indicated by dotted arrows. Chromosomal regions for potential deletions [relative read depth (RRD) of >0.9] are labeled with arrowheads. Approximate positions of three heterozygous *TT* genes are labeled with pale orange bars.
(TIF)

S5 Fig Proportion of MH sequence length at rejoined sites. Sizes (bp) are indicated at the bottom. Boundaries between ≤2 bp and ≥3 bp of MH sequences are indicated by dotted lines. Deletions having 2-bp MH sequences are reportedly the most common type of deletion in the M2 generation of gamma-irradiated wild-type Arabidopsis [10]. ** *p* < 0.01 (Fisher’s exact test).
(TIF)

S1 Table Summary of the mapping results. (XLSX)

S2 Table Mutations verified by PCR and Sanger sequencing. (XLSX)

S3 Table All mutations identified by M1 genome sequencing. (XLSX)
