# Supplementary figures and images for: Detection and characterization of genome-wide mutations in M1 vegetative cells of gamma-irradiated Arabidopsis
Source: PLoS Genet. 2022 Jan 20;18(1):e1009979. doi: 10.1371/journal.pgen.1009979 (PMC8775353; doi:10.1371/journal.pgen.1009979)

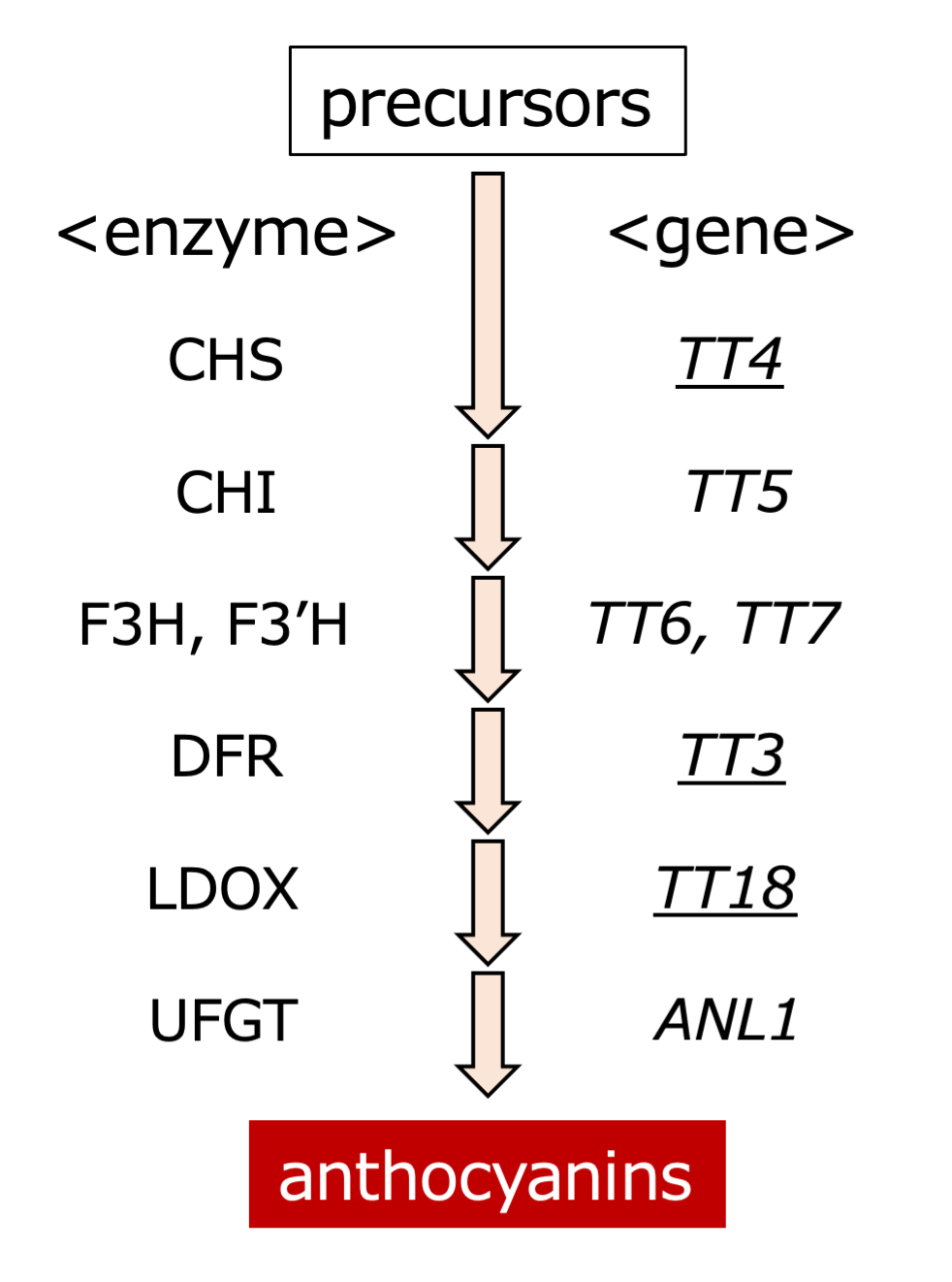

Supplement: S1 Fig — Anthocyanins are synthesized through sequential enzyme reactions involving chalcone synthase (CHS), chalcone isomerase (CHI), flavanone 3-hydroxylase (F3H), flavonoid 3’ hydroxylase (F3’H), dihydroflavonol 4-reductase (DFR), leucoanthocyanidin dioxygenase (LDOX), and UDP-glucose:flavonoid glucosyltransferase (UFGT). These enzymes are encoded by TRANSPARENT TESTA4 (TT4), TT5, TT6, TT7, TT3, TT18, and ANTHOCYANINLESS1 (ANL1), respectively. Three genes used in this study were underlined in right side of the pathway. (TIF) [file pgen.1009979.s001.tif]

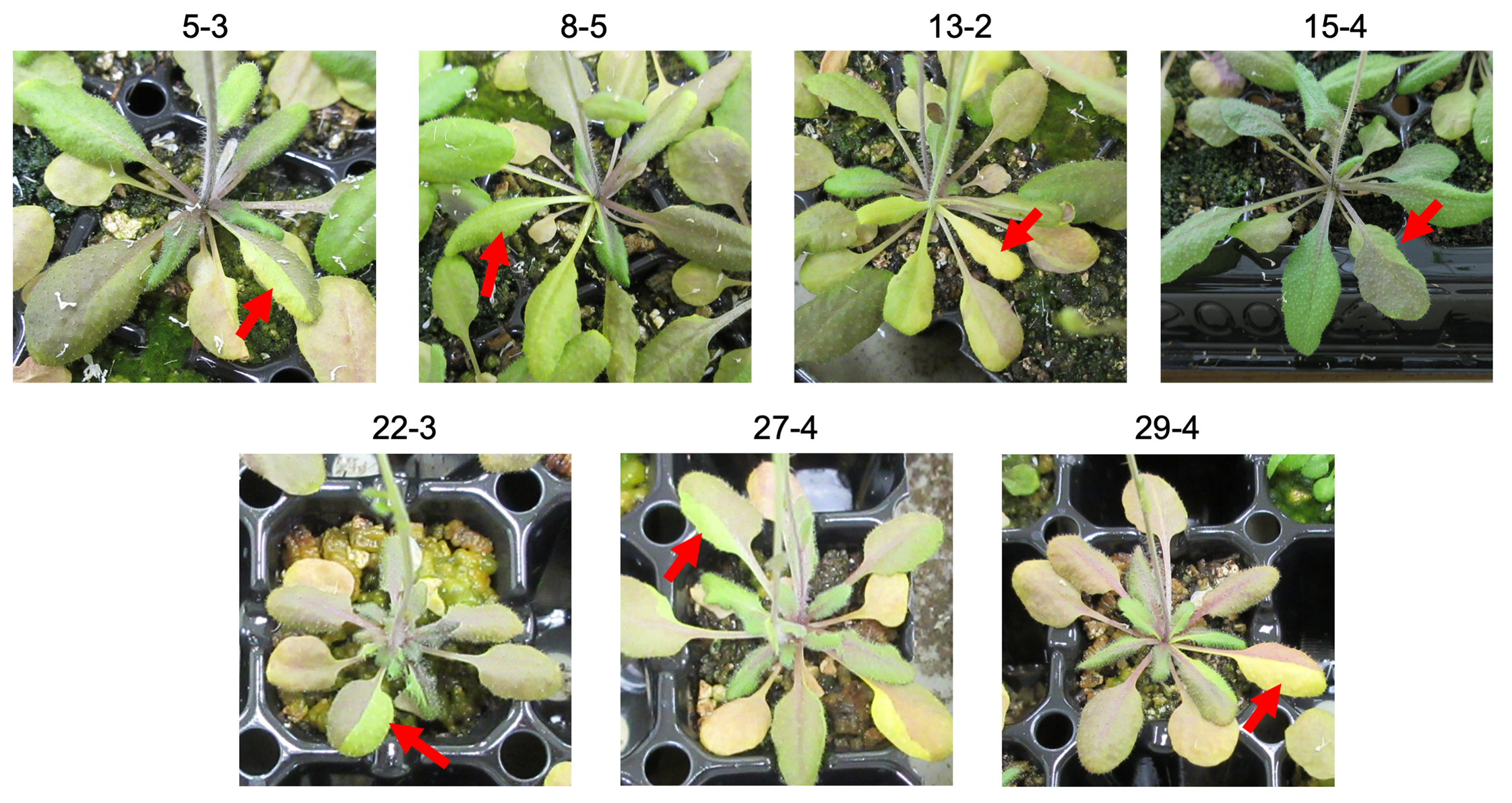

Supplement: S2 Fig — Red arrows indicate anthocyanin-less sectors analyzed by NGS. All plants were grown in 100 mm2 pots. Two plants (22–3 and 29–4) are also shown in Fig 1. (TIF) [file pgen.1009979.s002.tif]

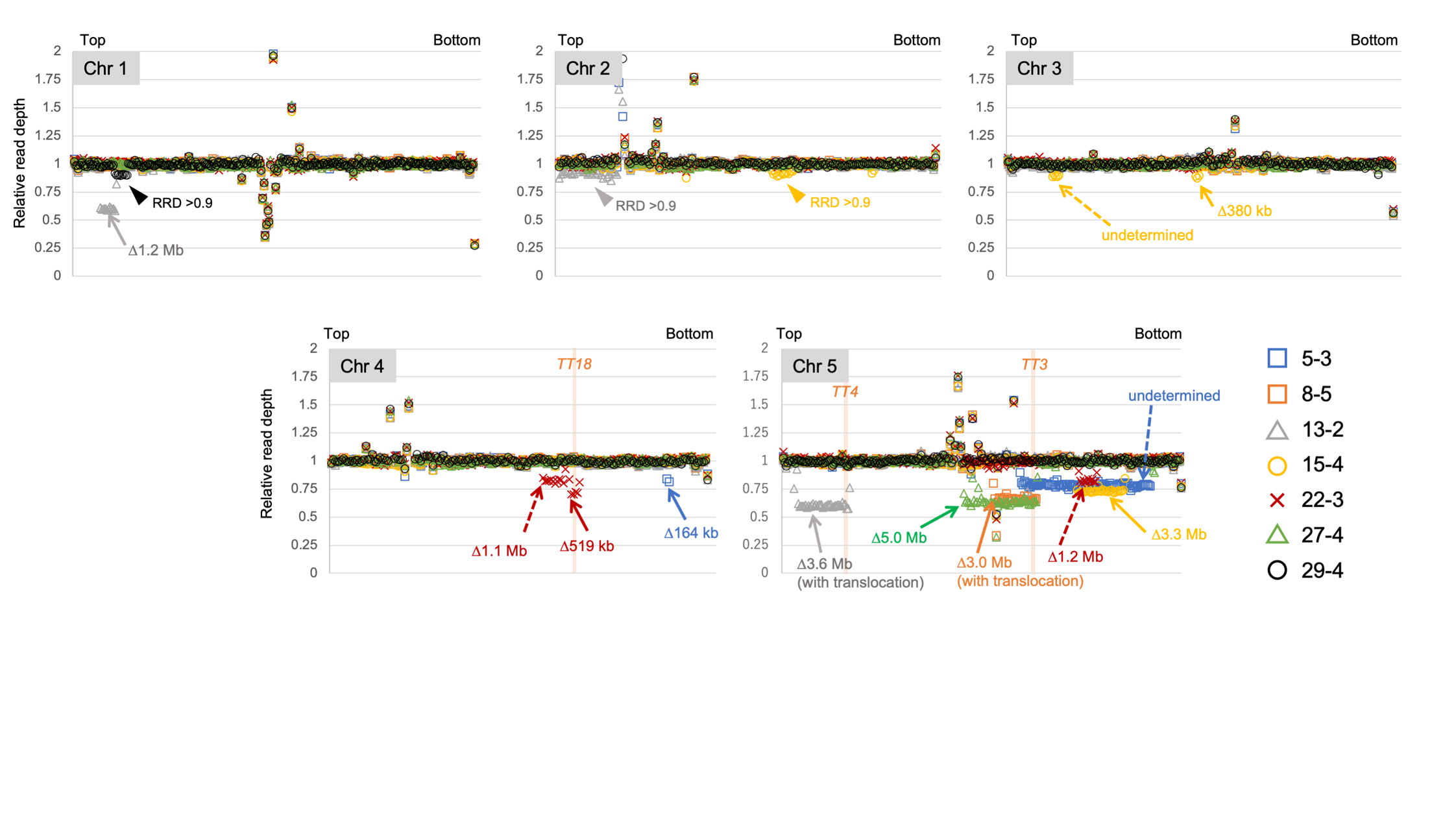

Supplement: S4 Fig — Eight ≥100 DEL candidates identified by the mutation-calling algorithms are indicated by solid arrows. Four additional ≥100 DEL candidates newly detected by this dosage analysis are indicated by dotted arrows. Chromosomal regions for potential deletions [relative read depth (RRD) of >0.9] are labeled with arrowheads. Approximate positions of three heterozygous TT genes are labeled with pale orange bars. (TIF) [file pgen.1009979.s004.tif]

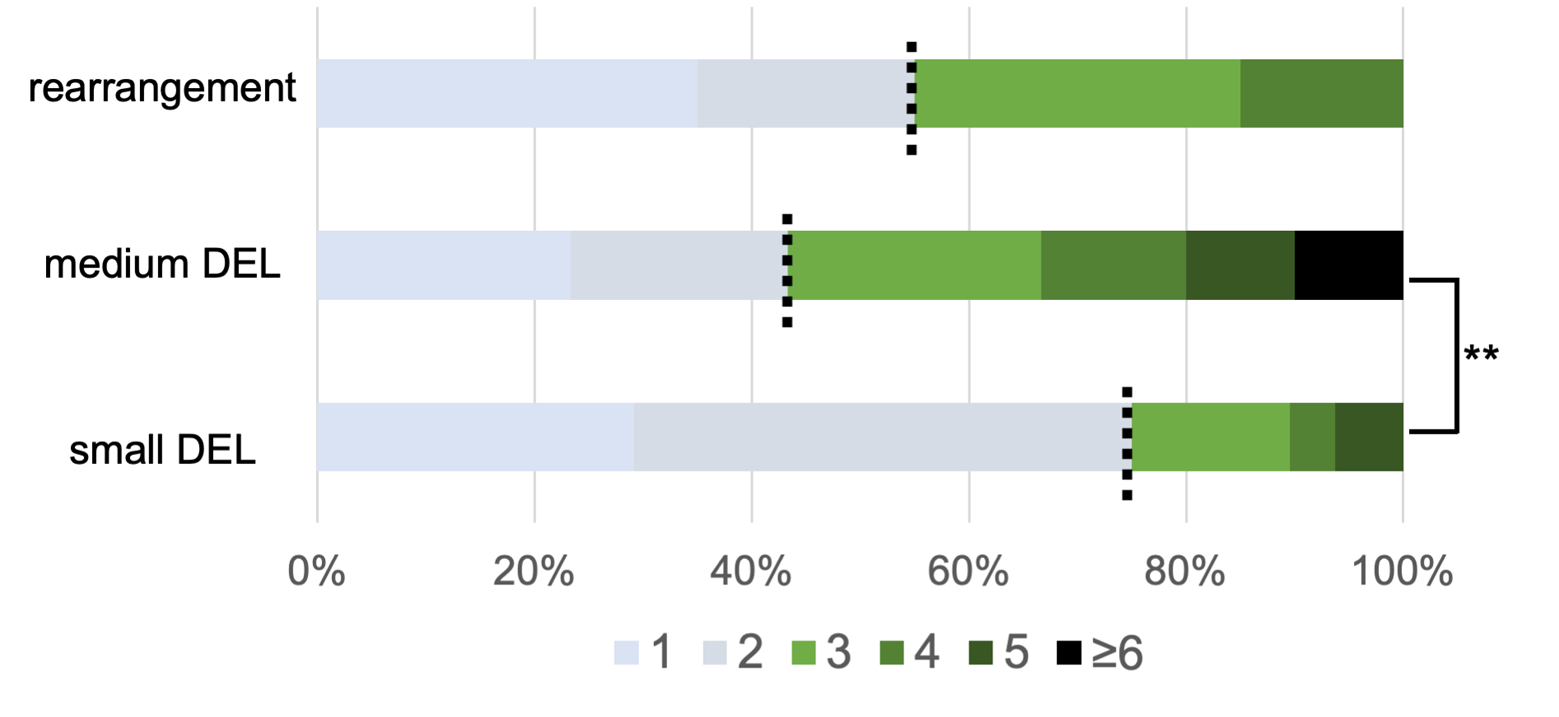

Supplement: S5 Fig — Sizes (bp) are indicated at the bottom. Boundaries between ≤2 bp and ≥3 bp of MH sequences are indicated by dotted lines. Deletions having 2-bp MH sequences are reportedly the most common type of deletion in the M2 generation of gamma-irradiated wild-type Arabidopsis [10]. ** p < 0.01 (Fisher’s exact test). (TIF) [file pgen.1009979.s005.tif]
